# 2,2′-Bis(meth­oxy­meth­oxy)-3-methyl-1,1′-binaphth­yl

**DOI:** 10.1107/S1600536811031722

**Published:** 2011-08-17

**Authors:** Rui M. B. Carrilho, Artur R. Abreu, Mariette M. Pereira, V. H. Rodrigues

**Affiliations:** a Chemistry Department, University of Coimbra, P-3004-516 Coimbra, Portugal; b CEMDRX, Physics Department, University of Coimbra, P-3004-516 Coimbra, Portugal

## Abstract

The title compound, C_25_H_24_O_4_, a meth­oxy­methyl (MOM) bis-protected BINOL derivative containing a methyl substituent in position 3, is a key inter­mediate for the synthesis of a great variety of chiral auxiliaries. The planes of the naphthyl aromatic rings are at an angle of 70.74 (3)°. There are no conventional hydrogen bonds binding the mol­ecules.

## Related literature

For the synthesis and catalytic applications of 3 and 3,3′-substituted BINOL derivatives, see: Shi & Wang (2002 ▶); Cox *et al.* (1992 ▶); Lingenfelter *et al.* (1981 ▶); Carrilho *et al.* (2009 ▶); Abreu *et al.* (2010 ▶). For the synthesis of the title compound, see: Cox *et al.* (1992 ▶). 
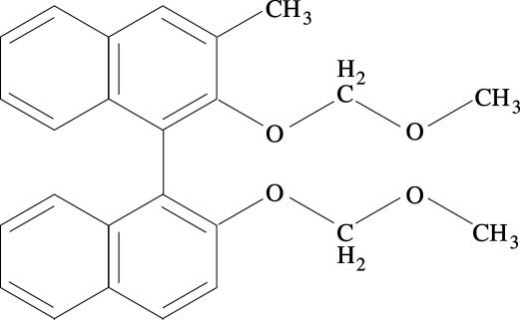


## Experimental

### 

#### Crystal data

C_25_H_24_O_4_
*M* *_r_* = 388.44
Orthorhombic, 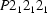

*a* = 8.1928 (3) Å
*b* = 14.3757 (5) Å
*c* = 17.1839 (6) Å
*V* = 2023.87 (12) Å^3^
*Z* = 4
Mo *K*α radiation
μ = 0.09 mm^−1^
*T* = 293 K
0.36 × 0.28 × 0.1 mm

#### Data collection

Bruker APEXII diffractometer
Absorption correction: multi-scan (*SADABS*; Sheldrick, 2003 ▶) *T*_min_ = 0.880, *T*_max_ = 1.000
30280 measured reflections
2046 independent reflections
1790 reflections with *I* > 2σ(*I*)
*R*_int_ = 0.050

#### Refinement

*R*[*F* ^2^ > 2σ(*F* ^2^)] = 0.031
*wR*(*F* ^2^) = 0.081
*S* = 1.05
2046 reflections
265 parameters
H-atom parameters constrained
Δρ_max_ = 0.09 e Å^−3^
Δρ_min_ = −0.11 e Å^−3^

### 

Data collection: *APEX2* (Bruker–Nonius, 2004 ▶); cell refinement: *SAINT* (Bruker, 2003 ▶); data reduction: *SAINT*; program(s) used to solve structure: *SHELXS97* (Sheldrick, 2008 ▶); program(s) used to refine structure: *SHELXL97* (Sheldrick, 2008 ▶); molecular graphics: *ORTEPII* (Johnson, 1976 ▶); software used to prepare material for publication: *SHELXL97*.

## Supplementary Material

Crystal structure: contains datablock(s) I, global. DOI: 10.1107/S1600536811031722/hg5065sup1.cif

Structure factors: contains datablock(s) I. DOI: 10.1107/S1600536811031722/hg5065Isup2.hkl

Supplementary material file. DOI: 10.1107/S1600536811031722/hg5065Isup3.cml

Additional supplementary materials:  crystallographic information; 3D view; checkCIF report

## Figures and Tables

**Fig. 1.**
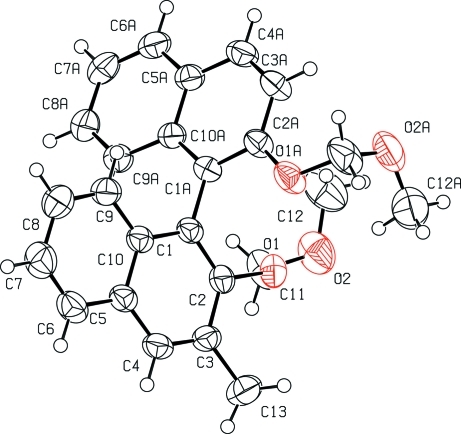
ORTEPII (Johnson, 1976) plot of the title compound. Displacement ellipsoids are drawn at the 50% level.

## supplementary crystallographic information

### Comment

The outcome of a given transition-metal catalyzed asymmetric transformation may depend on the steric and electronic properties of a chiral ligand. It is known that the ligand must have the symmetry and appropriate functionalities to discriminate the available space in the vicinity of the metal centre. In this context, 2,2'-binaphthol (BINOL) derivatives have generated particular interest because their modified backbone can influence not only the steric environment around the metal center but also the electronic properties of the oxygen atoms. Therefore, the strategic placement of substituents into the BINOL scaffold may lead to improved catalysts. In the early 1980's, Cram and co-workers synthesized a series of 3,3'-disubstituted BINOLs *via* Mannich intermediates and, in two diaryl cases, through Grignard cross-coupling reaction of 3,3'-dibromo-BINOL dimethyl ether and arylmagnesium bromides (Lingenfelter *et al.*, 1981). Later in the 1990's, Snieckus and co-workers described an efficient methodology to synthesize 3- and 3,3'-substituted 1,1'-bi-2-naphthols through directed *ortho*-metalation and Suzuki cross-coupling reactions (Cox *et al.*,1992).

Within our ongoing project of synthesizing BINOL derivatives (Carrilho *et al.* 2009, Abreu *et al.*, 2010), we obtained the title compound, C_25_H_24_O_4_, as a precursor of 3-substituted binaphthyl-based phosphorus ligands.

Single crystal X-ray diffraction shows that in the crystal structure of the title compound the planes of the naphthalene aromatic rings are at an angle of 70.74 (3)°. and that there are no conventional hydrogen bonds binding the molecules.

### Experimental

The title compound was synthesized from BINOL according to a slightly modified two step procedure, based on those previously reported (Shi & Wang, 2002, Cox *et al.*, 1992). First, under a nitrogen atmosphere, 1,1'-binaphthol (6.0 g, 21 mmol) was added to a suspension of NaH (3.4 g, 84 mmol) in anhydrous THF (60 ml) at 0°C, with stirring. This solution was stirred for 15 min, and then methoxymethyl chloride (4.0 ml, 53 mmol) was slowly added. The mixture was allowed to warm to room temperature and stirred for 5 h. After the standard procedures of quenching, washing and drying the organic layers, the solvent was removed and the compound 2,2'-bis(methoxymethoxy)-1,1'-binaphthyl was recrystallized from toluene/n-hexane. In the second step of the synthesis, under a nitrogen atmosphere, n-BuLi (1.6 *M* in hexene, 11.3 ml, 18 mmol) was added to a solution of 2,2'-bis(methoxymethoxy)-1,1'-binaphthyl (5.5 g, 15 mmol) in anhydrous THF (90 ml), at room temperature. The mixture was stirred for 4 h, which produced a grey suspension. After the mixture was cooled to 0°C, CH_3_I (1.2 ml, 19 mmol) was added. The reaction was allowed to warm to room temperature and stirred for 5 h. After quenching by a saturated solution of NH_4_Cl (50 ml), the aqueous layer was extracted with ethyl acetate (2× 50 ml) and the organic layers were combined and dried over Na_2_SO_4_. After removal of the solvent, the residue was purified by column chromatography on silica gel, using as eluent a mixture of n-hexane/ethyl acetate (10:1), which rendered the title compound (4.1 g, 70%). Crystals suitable for single-crystal X-ray diffraction were obtained after dissolution of the title compound (5 mg ml^-1^) in a mixture of n-hexane/ethyl acetate (10:1), and left open to air, at room temperature, for 36 h. The NMR data we obtained is in agreement with published values (Cox *et al.*, 1992).

^1^H NMR (CDCl_3_, TMS, 400 MHz) δ 2.58 (s, 3H, CH_3_), 2.89 (s, 3H, OCH_3_), 3.16 (s, 3H, OCH_3_), 4.55 (d, J=5.6 Hz, 1H, CH_2_), 4.64 (d, J=5.6 Hz, 1H, CH_2_), 5.01 (d, J=6.8 Hz, 1H, CH_2_), 5.12 (d, J=7.2 Hz, 1H, CH_2_), 7.12–7.36 (m, 6H, ArH), 7.57 (d, J=8.8 Hz, 1H, ArH), 7.80 (d, J=8.8 Hz, 2H, ArH), 7.86 (d, J=8.0 Hz, 1H, ArH), 7.95 (d, J=9.2 Hz, 1H, ArH). ^13^C NMR (CDCl_3_, TMS, 100 MHz) δ 17.9 (CH_3_), 55.9 (OCH_3_), 56.5 (OCH_3_), 95.0 (OCH_2_), 98.7 (OCH_2_), 116.7, 121.2, 124.1, 124.8, 125.1, 125.3, 125.7, 125.7, 126.6, 127.1, 127.8, 129.5, 129.7, 131.1, 131.6, 132.8, 134.1, 152.8, 153.1 (ArC).

### Refinement

All H atoms were were placed at idealized positions and refined as riding [C—H=0.93 (aromatic C), 0.97Å (CH_2_) and 0.96Å (CH_3_), *U*_iso_(H)=1.2*U*_eq_(C)].

The refined model structure is non-centrosymmetric with only atoms which are poor anomalous scatterers for the wavelength used, therefore Friedel pairs were merged before the final refinement. The meaningless Flack parameter obtained without merging of Friedel pairs was -0.3 (11). Absolute structure could not be reliably determined.

### Crystal data

**Table:**

C_25_H_24_O_4_	*F*(000) = 824
*M**_r_* = 388.44	*D*_x_ = 1.275 Mg m^−^^3^
Orthorhombic, *P*2_1_2_1_2_1_	Mo *K*α radiation, λ = 0.71073 Å
Hall symbol: P 2ac 2ab	Cell parameters from 6636 reflections
*a* = 8.1928 (3) Å	θ = 5.7–47.5°
*b* = 14.3757 (5) Å	µ = 0.09 mm^−^^1^
*c* = 17.1839 (6) Å	*T* = 293 K
*V* = 2023.87 (12) Å^3^	Prismatic, translucent colourless
*Z* = 4	0.36 × 0.28 × 0.1 mm

### Data collection

**Table:**

Bruker APEXII diffractometer	2046 independent reflections
Radiation source: fine-focus sealed tube	1790 reflections with *I* > 2σ(*I*)
graphite	*R*_int_ = 0.050
φ and ω scans	θ_max_ = 25.0°, θ_min_ = 3.7°
Absorption correction: multi-scan (*SADABS*; Sheldrick, 2003)	*h* = −9→9
*T*_min_ = 0.880, *T*_max_ = 1.000	*k* = −17→17
30280 measured reflections	*l* = −20→19

### Refinement

**Table:**

Refinement on *F*^2^	Primary atom site location: structure-invariant direct methods
Least-squares matrix: full	Secondary atom site location: difference Fourier map
*R*[*F*^2^ > 2σ(*F*^2^)] = 0.031	Hydrogen site location: inferred from neighbouring sites
*wR*(*F*^2^) = 0.081	H-atom parameters constrained
*S* = 1.05	*w* = 1/[σ^2^(*F*_o_^2^) + (0.0389*P*)^2^ + 0.3304*P*] where *P* = (*F*_o_^2^ + 2*F*_c_^2^)/3
2046 reflections	(Δ/σ)_max_ < 0.001
265 parameters	Δρ_max_ = 0.09 e Å^−^^3^
0 restraints	Δρ_min_ = −0.11 e Å^−^^3^

### Special details

**Table:**

Geometry. All e.s.d.'s (except the e.s.d. in the dihedral angle between two l.s. planes) are estimated using the full covariance matrix. The cell e.s.d.'s are taken into account individually in the estimation of e.s.d.'s in distances, angles and torsion angles; correlations between e.s.d.'s in cell parameters are only used when they are defined by crystal symmetry. An approximate (isotropic) treatment of cell e.s.d.'s is used for estimating e.s.d.'s involving l.s. planes.
Refinement. Refinement of *F*^2^ against ALL reflections. The weighted *R*-factor *wR* and goodness of fit *S* are based on *F*^2^, conventional *R*-factors *R* are based on *F*, with *F* set to zero for negative *F*^2^. The threshold expression of *F*^2^ > σ(*F*^2^) is used only for calculating *R*-factors(gt) *etc*. and is not relevant to the choice of reflections for refinement. *R*-factors based on *F*^2^ are statistically about twice as large as those based on *F*, and *R*- factors based on ALL data will be even larger.

### Fractional atomic coordinates and isotropic or equivalent isotropic displacement parameters (Å^2^)

**Table:**

	*x*	*y*	*z*	*U*_iso_*/*U*_eq_	
C1	0.7073 (2)	0.69011 (14)	0.84338 (12)	0.0369 (5)	
C2	0.6711 (2)	0.61570 (15)	0.89082 (13)	0.0405 (5)	
O1	0.73545 (19)	0.52875 (10)	0.87448 (9)	0.0491 (4)	
C11	0.6355 (3)	0.47209 (18)	0.82486 (19)	0.0677 (7)	
H11A	0.5365	0.4542	0.8519	0.081*	
H11B	0.6053	0.5066	0.7786	0.081*	
O2	0.7235 (3)	0.39398 (12)	0.80452 (14)	0.0821 (6)	
C12	0.8311 (5)	0.4088 (2)	0.7413 (2)	0.0951 (11)	
H12A	0.9070	0.4572	0.7544	0.143*	
H12B	0.7695	0.4268	0.6962	0.143*	
H12C	0.8896	0.3524	0.7305	0.143*	
C3	0.5780 (3)	0.62609 (16)	0.96027 (12)	0.0443 (5)	
C13	0.5472 (4)	0.54406 (19)	1.01259 (16)	0.0659 (7)	
H13A	0.4833	0.5635	1.0565	0.099*	
H13B	0.6495	0.5194	1.0304	0.099*	
H13C	0.4892	0.4969	0.9843	0.099*	
C4	0.5214 (3)	0.71246 (16)	0.97842 (12)	0.0459 (5)	
H4	0.4591	0.7199	1.0232	0.055*	
C5	0.5535 (3)	0.79062 (15)	0.93211 (12)	0.0415 (5)	
C6	0.4929 (3)	0.88020 (17)	0.95132 (14)	0.0549 (6)	
H6	0.4272	0.8875	0.9950	0.066*	
C7	0.5291 (4)	0.95506 (18)	0.90699 (15)	0.0628 (7)	
H7	0.4878	1.0132	0.9203	0.075*	
C8	0.6284 (3)	0.94558 (16)	0.84124 (15)	0.0568 (6)	
H8	0.6545	0.9977	0.8117	0.068*	
C9	0.6871 (3)	0.86076 (15)	0.82018 (13)	0.0466 (5)	
H9	0.7526	0.8556	0.7762	0.056*	
C10	0.6502 (2)	0.78014 (14)	0.86413 (11)	0.0382 (5)	
C1A	0.8059 (3)	0.67661 (14)	0.77123 (12)	0.0387 (5)	
C2A	0.9703 (3)	0.65712 (15)	0.77629 (12)	0.0434 (5)	
O1A	1.03316 (19)	0.65245 (13)	0.85048 (9)	0.0558 (5)	
C11A	1.1991 (3)	0.6266 (2)	0.86092 (17)	0.0720 (8)	
H11C	1.2675	0.6680	0.8306	0.086*	
H11D	1.2280	0.6345	0.9153	0.086*	
O2A	1.2315 (3)	0.53575 (18)	0.83918 (12)	0.0843 (7)	
C12A	1.1601 (5)	0.4669 (3)	0.8900 (2)	0.0956 (11)	
H12D	1.0433	0.4700	0.8865	0.143*	
H12E	1.1933	0.4788	0.9427	0.143*	
H12F	1.1964	0.4061	0.8747	0.143*	
C3A	1.0660 (3)	0.64502 (17)	0.70922 (14)	0.0515 (6)	
H3A	1.1769	0.6324	0.7138	0.062*	
C4A	0.9966 (3)	0.65178 (15)	0.63747 (14)	0.0501 (6)	
H4A	1.0611	0.6431	0.5935	0.060*	
C5A	0.8289 (3)	0.67162 (14)	0.62827 (12)	0.0430 (5)	
C6A	0.7546 (3)	0.67856 (15)	0.55421 (13)	0.0504 (6)	
H6A	0.8170	0.6687	0.5098	0.061*	
C7A	0.5940 (4)	0.69931 (17)	0.54687 (13)	0.0559 (6)	
H7A	0.5472	0.7036	0.4977	0.067*	
C8A	0.4987 (3)	0.71424 (16)	0.61322 (13)	0.0532 (6)	
H8A	0.3890	0.7296	0.6079	0.064*	
C9A	0.5650 (3)	0.70648 (16)	0.68543 (13)	0.0470 (5)	
H9A	0.4992	0.7155	0.7289	0.056*	
C10A	0.7327 (3)	0.68483 (13)	0.69579 (11)	0.0390 (5)	

### Atomic displacement parameters (Å^2^)

**Table:**

	*U*^11^	*U*^22^	*U*^33^	*U*^12^	*U*^13^	*U*^23^
C1	0.0289 (10)	0.0477 (11)	0.0339 (10)	0.0011 (9)	−0.0006 (9)	−0.0009 (8)
C2	0.0300 (10)	0.0492 (12)	0.0424 (11)	0.0032 (9)	−0.0034 (9)	0.0026 (9)
O1	0.0449 (8)	0.0436 (8)	0.0588 (9)	0.0070 (7)	−0.0041 (8)	0.0015 (7)
C11	0.0478 (14)	0.0565 (14)	0.099 (2)	0.0006 (12)	0.0053 (15)	−0.0138 (15)
O2	0.0777 (14)	0.0468 (9)	0.1218 (17)	0.0008 (10)	0.0153 (13)	−0.0097 (11)
C12	0.087 (2)	0.095 (2)	0.104 (3)	0.006 (2)	0.020 (2)	−0.026 (2)
C3	0.0374 (11)	0.0582 (13)	0.0371 (11)	−0.0009 (11)	−0.0004 (9)	0.0092 (10)
C13	0.0686 (17)	0.0701 (16)	0.0589 (14)	−0.0037 (15)	0.0040 (14)	0.0218 (13)
C4	0.0381 (12)	0.0654 (14)	0.0341 (11)	0.0012 (11)	0.0035 (10)	0.0004 (10)
C5	0.0346 (11)	0.0536 (12)	0.0364 (11)	0.0024 (10)	−0.0002 (9)	−0.0019 (9)
C6	0.0525 (14)	0.0616 (14)	0.0504 (13)	0.0105 (13)	0.0064 (12)	−0.0105 (11)
C7	0.0711 (18)	0.0503 (13)	0.0670 (16)	0.0112 (14)	0.0031 (15)	−0.0087 (12)
C8	0.0632 (16)	0.0457 (13)	0.0614 (14)	−0.0023 (12)	−0.0015 (14)	0.0038 (11)
C9	0.0456 (13)	0.0494 (12)	0.0448 (12)	−0.0019 (11)	0.0045 (11)	0.0022 (9)
C10	0.0314 (10)	0.0468 (11)	0.0366 (10)	−0.0002 (9)	−0.0020 (9)	−0.0007 (9)
C1A	0.0355 (11)	0.0413 (10)	0.0393 (11)	−0.0011 (9)	0.0053 (9)	−0.0023 (9)
C2A	0.0347 (11)	0.0528 (12)	0.0427 (12)	0.0004 (10)	0.0013 (10)	−0.0056 (10)
O1A	0.0343 (8)	0.0873 (12)	0.0457 (9)	0.0087 (8)	−0.0035 (7)	−0.0110 (8)
C11A	0.0332 (13)	0.117 (2)	0.0653 (17)	0.0078 (15)	−0.0092 (13)	−0.0093 (17)
O2A	0.0643 (12)	0.1207 (18)	0.0678 (12)	0.0428 (13)	0.0019 (11)	−0.0101 (13)
C12A	0.095 (3)	0.109 (3)	0.083 (2)	0.032 (2)	0.003 (2)	0.003 (2)
C3A	0.0365 (12)	0.0641 (14)	0.0541 (14)	0.0042 (11)	0.0086 (12)	−0.0077 (11)
C4A	0.0505 (14)	0.0532 (13)	0.0465 (13)	0.0024 (11)	0.0157 (11)	−0.0033 (10)
C5A	0.0496 (13)	0.0383 (10)	0.0412 (12)	−0.0012 (10)	0.0076 (10)	0.0015 (9)
C6A	0.0668 (16)	0.0452 (12)	0.0394 (11)	0.0001 (12)	0.0096 (12)	0.0042 (9)
C7A	0.0724 (18)	0.0548 (14)	0.0405 (12)	0.0009 (13)	−0.0085 (13)	0.0079 (10)
C8A	0.0502 (13)	0.0586 (13)	0.0508 (13)	0.0054 (12)	−0.0053 (12)	0.0074 (11)
C9A	0.0438 (12)	0.0550 (13)	0.0422 (12)	0.0019 (11)	0.0008 (10)	0.0017 (10)
C10A	0.0411 (11)	0.0384 (10)	0.0375 (11)	−0.0014 (9)	0.0038 (9)	0.0009 (8)

### Geometric parameters (Å, °)

**Table:**

C1—C2	1.377 (3)	C9—C10	1.416 (3)
C1—C10	1.422 (3)	C9—H9	0.9300
C1—C1A	1.493 (3)	C1A—C2A	1.379 (3)
C2—O1	1.385 (2)	C1A—C10A	1.433 (3)
C2—C3	1.424 (3)	C2A—O1A	1.376 (3)
O1—C11	1.435 (3)	C2A—C3A	1.405 (3)
C11—O2	1.379 (3)	O1A—C11A	1.421 (3)
C11—H11A	0.9700	C11A—O2A	1.384 (4)
C11—H11B	0.9700	C11A—H11C	0.9700
O2—C12	1.415 (4)	C11A—H11D	0.9700
C12—H12A	0.9600	O2A—C12A	1.444 (4)
C12—H12B	0.9600	C12A—H12D	0.9600
C12—H12C	0.9600	C12A—H12E	0.9600
C3—C4	1.362 (3)	C12A—H12F	0.9600
C3—C13	1.504 (3)	C3A—C4A	1.361 (3)
C13—H13A	0.9600	C3A—H3A	0.9300
C13—H13B	0.9600	C4A—C5A	1.412 (3)
C13—H13C	0.9600	C4A—H4A	0.9300
C4—C5	1.402 (3)	C5A—C6A	1.414 (3)
C4—H4	0.9300	C5A—C10A	1.415 (3)
C5—C10	1.419 (3)	C6A—C7A	1.355 (4)
C5—C6	1.419 (3)	C6A—H6A	0.9300
C6—C7	1.352 (4)	C7A—C8A	1.399 (4)
C6—H6	0.9300	C7A—H7A	0.9300
C7—C8	1.399 (4)	C8A—C9A	1.359 (3)
C7—H7	0.9300	C8A—H8A	0.9300
C8—C9	1.360 (3)	C9A—C10A	1.420 (3)
C8—H8	0.9300	C9A—H9A	0.9300
			
C2—C1—C10	119.19 (18)	C9—C10—C5	118.11 (19)
C2—C1—C1A	120.49 (18)	C9—C10—C1	122.76 (18)
C10—C1—C1A	120.32 (18)	C5—C10—C1	119.12 (18)
C1—C2—O1	119.92 (18)	C2A—C1A—C10A	118.88 (19)
C1—C2—C3	122.00 (19)	C2A—C1A—C1	120.19 (19)
O1—C2—C3	117.93 (18)	C10A—C1A—C1	120.93 (17)
C2—O1—C11	114.54 (17)	O1A—C2A—C1A	115.70 (19)
O2—C11—O1	108.3 (2)	O1A—C2A—C3A	123.05 (19)
O2—C11—H11A	110.0	C1A—C2A—C3A	121.2 (2)
O1—C11—H11A	110.0	C2A—O1A—C11A	119.18 (19)
O2—C11—H11B	110.0	O2A—C11A—O1A	113.3 (2)
O1—C11—H11B	110.0	O2A—C11A—H11C	108.9
H11A—C11—H11B	108.4	O1A—C11A—H11C	108.9
C11—O2—C12	113.4 (2)	O2A—C11A—H11D	108.9
O2—C12—H12A	109.5	O1A—C11A—H11D	108.9
O2—C12—H12B	109.5	H11C—C11A—H11D	107.7
H12A—C12—H12B	109.5	C11A—O2A—C12A	114.0 (2)
O2—C12—H12C	109.5	O2A—C12A—H12D	109.5
H12A—C12—H12C	109.5	O2A—C12A—H12E	109.5
H12B—C12—H12C	109.5	H12D—C12A—H12E	109.5
C4—C3—C2	118.03 (19)	O2A—C12A—H12F	109.5
C4—C3—C13	121.4 (2)	H12D—C12A—H12F	109.5
C2—C3—C13	120.6 (2)	H12E—C12A—H12F	109.5
C3—C13—H13A	109.5	C4A—C3A—C2A	120.1 (2)
C3—C13—H13B	109.5	C4A—C3A—H3A	120.0
H13A—C13—H13B	109.5	C2A—C3A—H3A	120.0
C3—C13—H13C	109.5	C3A—C4A—C5A	121.5 (2)
H13A—C13—H13C	109.5	C3A—C4A—H4A	119.3
H13B—C13—H13C	109.5	C5A—C4A—H4A	119.3
C3—C4—C5	122.5 (2)	C4A—C5A—C6A	122.3 (2)
C3—C4—H4	118.8	C4A—C5A—C10A	118.5 (2)
C5—C4—H4	118.8	C6A—C5A—C10A	119.3 (2)
C4—C5—C10	119.15 (19)	C7A—C6A—C5A	121.1 (2)
C4—C5—C6	122.0 (2)	C7A—C6A—H6A	119.4
C10—C5—C6	118.9 (2)	C5A—C6A—H6A	119.4
C7—C6—C5	121.0 (2)	C6A—C7A—C8A	120.0 (2)
C7—C6—H6	119.5	C6A—C7A—H7A	120.0
C5—C6—H6	119.5	C8A—C7A—H7A	120.0
C6—C7—C8	120.4 (2)	C9A—C8A—C7A	120.6 (2)
C6—C7—H7	119.8	C9A—C8A—H8A	119.7
C8—C7—H7	119.8	C7A—C8A—H8A	119.7
C9—C8—C7	120.5 (2)	C8A—C9A—C10A	121.3 (2)
C9—C8—H8	119.7	C8A—C9A—H9A	119.4
C7—C8—H8	119.7	C10A—C9A—H9A	119.4
C8—C9—C10	121.1 (2)	C5A—C10A—C9A	117.73 (19)
C8—C9—H9	119.5	C5A—C10A—C1A	119.81 (19)
C10—C9—H9	119.5	C9A—C10A—C1A	122.45 (19)
			
C10—C1—C2—O1	−175.41 (18)	C10—C1—C1A—C2A	108.5 (2)
C1A—C1—C2—O1	4.3 (3)	C2—C1—C1A—C10A	109.6 (2)
C10—C1—C2—C3	0.0 (3)	C10—C1—C1A—C10A	−70.6 (3)
C1A—C1—C2—C3	179.70 (19)	C10A—C1A—C2A—O1A	178.49 (18)
C1—C2—O1—C11	−90.4 (2)	C1—C1A—C2A—O1A	−0.7 (3)
C3—C2—O1—C11	94.1 (2)	C10A—C1A—C2A—C3A	−0.2 (3)
C2—O1—C11—O2	171.5 (2)	C1—C1A—C2A—C3A	−179.4 (2)
O1—C11—O2—C12	−82.8 (3)	C1A—C2A—O1A—C11A	175.8 (2)
C1—C2—C3—C4	1.4 (3)	C3A—C2A—O1A—C11A	−5.5 (4)
O1—C2—C3—C4	176.8 (2)	C2A—O1A—C11A—O2A	−66.0 (3)
C1—C2—C3—C13	−177.2 (2)	O1A—C11A—O2A—C12A	−69.6 (3)
O1—C2—C3—C13	−1.7 (3)	O1A—C2A—C3A—C4A	−179.1 (2)
C2—C3—C4—C5	−0.9 (3)	C1A—C2A—C3A—C4A	−0.5 (4)
C13—C3—C4—C5	177.6 (2)	C2A—C3A—C4A—C5A	0.5 (4)
C3—C4—C5—C10	−0.8 (3)	C3A—C4A—C5A—C6A	−179.8 (2)
C3—C4—C5—C6	179.5 (2)	C3A—C4A—C5A—C10A	0.2 (3)
C4—C5—C6—C7	178.1 (2)	C4A—C5A—C6A—C7A	−178.8 (2)
C10—C5—C6—C7	−1.6 (4)	C10A—C5A—C6A—C7A	1.2 (3)
C5—C6—C7—C8	−0.4 (4)	C5A—C6A—C7A—C8A	0.0 (4)
C6—C7—C8—C9	1.3 (4)	C6A—C7A—C8A—C9A	−1.2 (4)
C7—C8—C9—C10	−0.3 (4)	C7A—C8A—C9A—C10A	1.2 (4)
C8—C9—C10—C5	−1.6 (3)	C4A—C5A—C10A—C9A	178.8 (2)
C8—C9—C10—C1	179.1 (2)	C6A—C5A—C10A—C9A	−1.2 (3)
C4—C5—C10—C9	−177.2 (2)	C4A—C5A—C10A—C1A	−0.9 (3)
C6—C5—C10—C9	2.5 (3)	C6A—C5A—C10A—C1A	179.07 (19)
C4—C5—C10—C1	2.1 (3)	C8A—C9A—C10A—C5A	0.0 (3)
C6—C5—C10—C1	−178.2 (2)	C8A—C9A—C10A—C1A	179.7 (2)
C2—C1—C10—C9	177.6 (2)	C2A—C1A—C10A—C5A	0.9 (3)
C1A—C1—C10—C9	−2.2 (3)	C1—C1A—C10A—C5A	−179.94 (19)
C2—C1—C10—C5	−1.7 (3)	C2A—C1A—C10A—C9A	−178.8 (2)
C1A—C1—C10—C5	178.57 (18)	C1—C1A—C10A—C9A	0.4 (3)
C2—C1—C1A—C2A	−71.2 (3)		
